# Bilobed flap for lower lip reconstruction: A two-case series and technical considerations

**DOI:** 10.1016/j.bjorl.2025.101738

**Published:** 2025-11-28

**Authors:** Victor Bandini Vieira, Giovana Cruz Corsi, Rafael Pereira de Souza, Rafael De Cicco

**Affiliations:** Instituto de Cancer Dr. Arnaldo, Departamento de Cirurgia de Cabeça e Pescoço, São Paulo, SP, Brazil

**Keywords:** Head and neck surgery, Lip neoplasms, Reconstructive, surgical procedures

## Introduction

Lower lip reconstruction is a challenging surgical task, particularly after oncologic resections. Restoring oral competence, mobility, and aesthetics is critical to patient quality of life. Among available techniques, the bilobed flap has emerged as a versatile and effective option for moderate lower lip defects.^1^

This flap involves the sequential transposition of two adjacent lobes, allowing anatomical closure with redistributed tension and minimal distortion. While bilobed flaps are widely employed in facial reconstruction, their specific application to the lower lip remains underreported.^2^ Their use is especially beneficial in preserving vascularity, minimizing complications like microstomia, and optimizing functional and aesthetic outcomes.^1^^,^^2^

## Case reports

Case 1 A 65-year-old male, ex-smoker, presented with a central lower lip ulcer measuring 3.5 cm, impairing speech and feeding. Staged as cT2N0M0, he underwent resection followed by reconstruction using a local advancement flap. Histopathology revealed invasive squamous cell carcinoma (pT3N0M0) with muscle invasion and perineural spread.

**Fig. 1:**
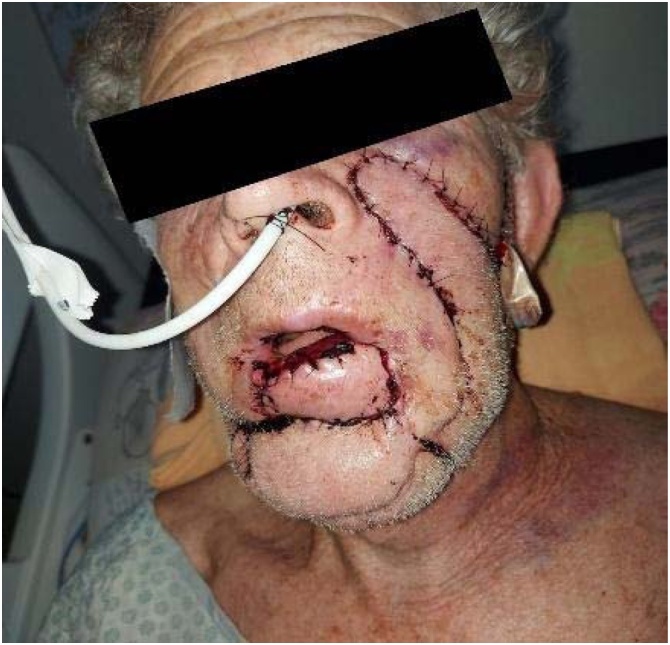
Immediate postoperative period, showing the bilobed flap designed based on the left side of the face.

**Fig. 2:**
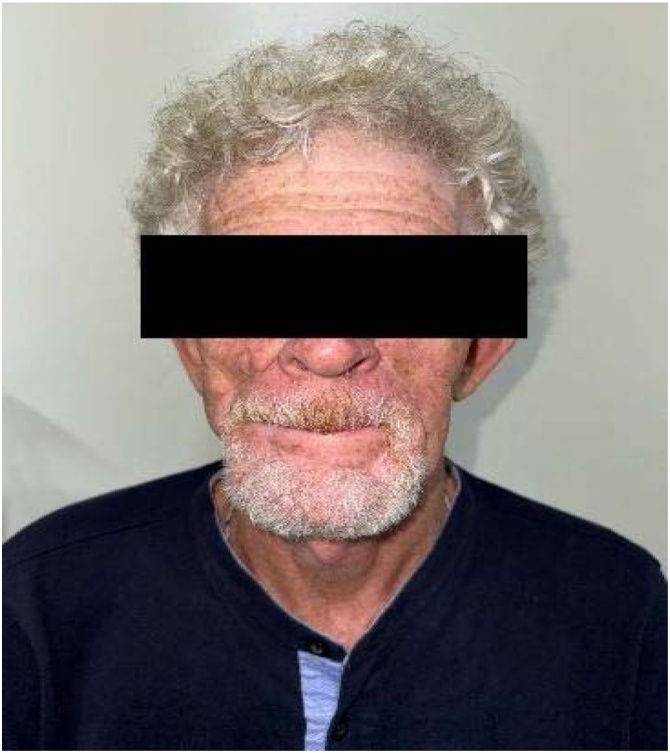
Result 24 months after bilobed flap rotation.

The advancement flap developed partial necrosis. A secondary nasogenian flap failed due to distal ischemia, compromising oral seal. A third submental flap provided tissue replacement but failed functionally. Sixteen months later, a bilobed flap based on the left cheek was performed. This final reconstruction restored oral competence and aesthetics, with stable integration and no recurrence at 24-month follow-up (Fig. 1, Fig. 2).

**Fig. 3:**
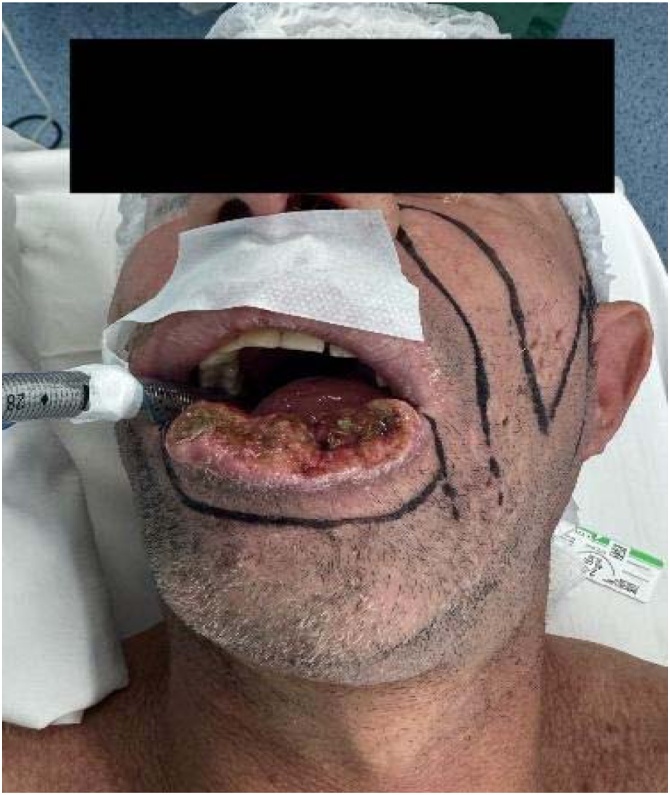
A chronic ulcerative lesion on the lower lip, cT2N0M0.

Case 2 A 47-year-old smoker presented with a chronic 3.0 cm ulcer on the mid-lower lip. Staging was cT2N0M0. Segmental resection was followed by immediate bilobed flap reconstruction based on the right cheek. The primary lobe covered the defect, and the secondary closed the donor site, preserving symmetry and function (Fig. 3).

**Fig. 4:**
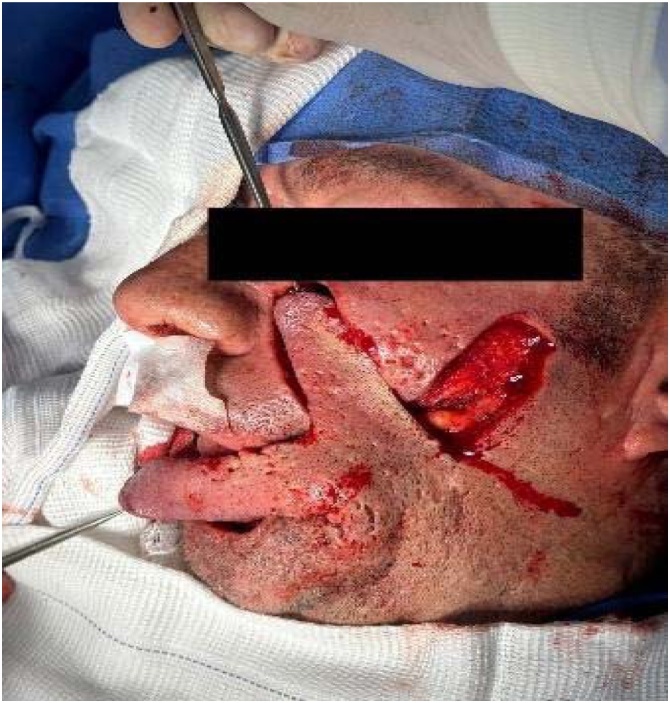
Segmental resection of the lower lip, followed by immediate reconstruction with a bilobed flap based on the right cheek.

**Fig. 5:**
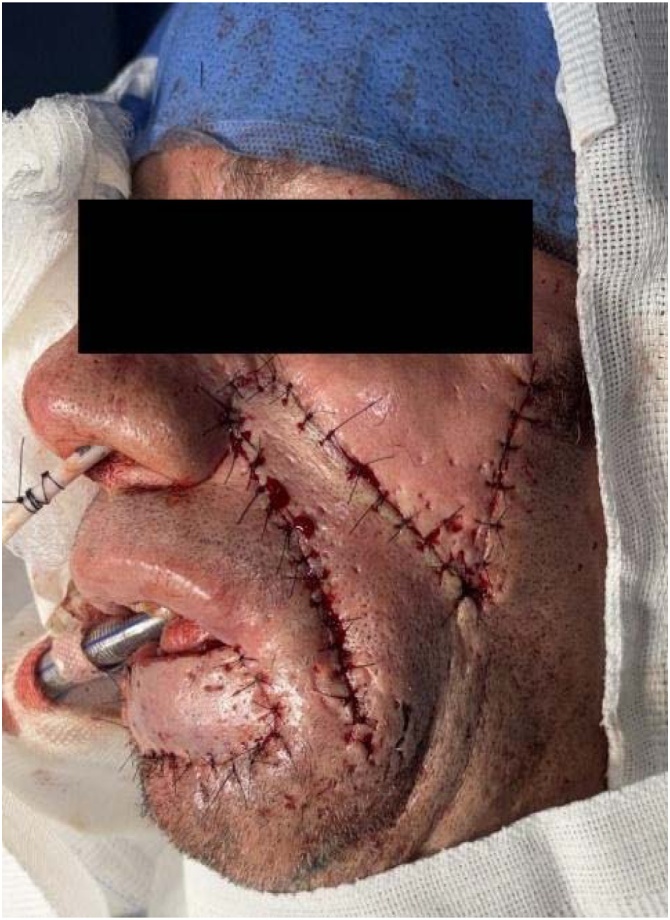
Final result after closing without tension.

Histopathology confirmed keratinizing squamous cell carcinoma (pT3N0M0) with muscle invasion and perineural spread. The postoperative course was uneventful, with excellent healing, oral competence, and no recurrence at 12-months (Fig. 4, Fig. 5).

## Discussion

Reconstruction of the lower lip demands restoration of its sphincter mechanism, mobility, and cosmesis. The bilobed flap is particularly suited to moderate defects not involving the commissures. Its geometric configuration allows for efficient tension redistribution, sparing adjacent anatomical structures.^3^

Unlike advancement or nasogenian flaps, which can create vertical traction on the upper lip, the bilobed flap uses genian tissue to preserve contour. The secondary lobe is strategically placed to redirect closure tension laterally, enhancing perioral symmetry.^1^

The flap provides ideal tissue match in terms of thickness, pliability, and color. As seen in the second case, immediate reconstruction preserved oral competence and avoided complications like retraction or microstomia.

Vascularity in the cheek is robust, supplied by branches of the facial artery. The flap can function as either a random or axial-pattern flap, incorporating perforators from the angular or facial arteries when designed appropriately.^4^ This was key in Case 1, where prior failures gave way to successful integration upon delayed bilobed reconstruction.

Originally described by Esser in 1918 and later adapted for lip reconstruction by Fujimori, the bilobed flap has proven reliable in various facial areas.^5^ Studies, including Tissiani et al., report high success rates and low complications, supporting its value in facial reconstruction.^1^

Its proximity to the defect also minimizes donor site morbidity, avoiding distant flaps or grafts, reducing operative time, and preventing secondary deformities.^1^^,^^2^

Technically, careful planning is essential maintaining a 1:1 ratio between base and length, and minimizing distal tension. Proper arc and lobe dimensions ensure perfusion and optimal contour. Our cases reinforce its success when executed with precision.

## Conclusion

The bilobed flap is a reliable, low-morbidity option for lower lip reconstruction in moderate defects. It offers excellent functional and aesthetic outcomes, with preserved oral competence and minimal distortion. Both immediate and delayed applications in our cases confirm its value in head and neck reconstructive surgery.

## ORCID IDs

Victor Bandini Vieira: 0009-0004-0529-9141

Giovana Cruz Corsi: 0000-0002-7822-2020

Rafael Pereira de Souza: 0000-0001-5916-9730

Rafael de Cicco: 0000-0003-2505-0461

## Funding

None.

## Declaration of competing interest

The authors declare no conflicts of interest.
